# Identification of *Trypanosoma cruzi* Polyamine Transport Inhibitors by Computational Drug Repurposing

**DOI:** 10.3389/fmed.2019.00256

**Published:** 2019-11-08

**Authors:** Chantal Reigada, Melisa Sayé, Otto Phanstiel, Edward Valera-Vera, Mariana R. Miranda, Claudio A. Pereira

**Affiliations:** ^1^Universidad de Buenos Aires, Facultad de Medicina, Instituto de Investigaciones Médicas A. Lanari, Buenos Aires, Argentina; ^2^Consejo Nacional de Investigaciones Científicas y Técnicas, Laboratorio de Parasitología Molecular, Instituto de Investigaciones Médicas (IDIM), Universidad de Buenos Aires, Buenos Aires, Argentina; ^3^Department of Medical Education, University of Central Florida, Orlando, FL, United States

**Keywords:** *Trypanosoma cruzi*, Chagas disease, polyamine transport, drug repositioning, trypanocidal drugs, polyamines

## Abstract

*Trypanosoma cruzi* is the causative agent of Chagas disease, a parasitic infection endemic in Latin America. In *T. cruzi* the transport of polyamines is essential because this organism is unable to synthesize these compounds *de novo*. Therefore, the uptake of polyamines from the extracellular medium is critical for survival of the parasite. The anthracene-putrescine conjugate Ant4 was first designed as a polyamine transport probe in cancer cells. Ant4 was also found to inhibit the polyamine transport system and produced a strong trypanocidal effect in *T. cruzi*. Considering that Ant4 is not currently approved by the FDA, in this work we performed computer simulations to find trypanocidal drugs approved for use in humans that have structures and activities similar to Ant4. Through a similarity ligand-based virtual screening using Ant4 as reference molecule, four possible inhibitors of polyamine transport were found. Three of them, promazine, chlorpromazine, and clomipramine, showed to be effective inhibitors of putrescine uptake, and also revealed a high trypanocidal activity against *T. cruzi* amastigotes (IC_50_ values of 3.8, 1.9, and 2.9 μM, respectively) and trypomastigotes (IC_50_ values of 3.4, 2.7, and 1.3 μM, respectively) while in epimastigotes the IC_50_ were significantly higher (34.7, 41.4, and 39.7 μM, respectively). Finally, molecular docking simulations suggest that the interactions between the *T. cruzi* polyamine transporter TcPAT12 and all the identified inhibitors occur in the same region of the protein. However, this location is different from the site occupied by the natural substrates. The value of this effort is that repurposing known drugs in the treatment of other pathologies, especially neglected diseases such as Chagas disease, significantly decreases the time and economic cost of implementation.

## Introduction

The protozoan *Trypanosoma cruzi* is the causative agent of Chagas disease, a severe parasitic infection endemic in Latin America with ~7 million infected and more than 70 million people at risk, mostly living in conditions of extreme poverty (1, 2). With no immediate prospect of a vaccine, developing therapeutic alternatives to treat Chagas disease is an urgent need. The available drugs, benznidazole, and nifurtimox, have been used for nearly half a century and can cause severe side effects decreasing the quality of life of the patients. In addition, they are only partially effective in the treatment of the chronic phase of the disease, when most of the patients are diagnosed (3). For example, the recent results of the trial “BENznidazole Evaluation For Interrupting Trypanosomiasis” (BENEFIT) showed that this drug does not produce any improvement on the Chagasic cardiopathy in patients in the chronic phase of the disease, highlighting the necessity for the development of new treatments (4).

Polyamines are aliphatic polycations, which are present in all living organisms. One of the most interesting chemical features of polyamines is the regularly spaced positive charges and their ability to form ionic interactions with other molecules (5). These low-molecular weight compounds are essential for cell survival because they are involved in a wide variety of metabolic processes. In this sense, the most abundant polyamines in nature, putrescine, spermidine, and spermine participate in cell growth and proliferation, signal transduction, gene transcription, and translation processes (6).

In *T. cruzi* the uptake of polyamines is essential for cell survival because the parasite is not able to synthesize polyamines *de novo* due to the lack of the enzymes arginine decarboxylase and ornithine decarboxylase. Unlike mammals, *T. cruzi* only obtains polyamines from the extracellular medium by transport processes (7, 8). The permease called TcPAT12 (also known as TcPOT1) is until now the only functionally validated polyamine transporter in *T. cruzi* (9, 10). This protein constitutes a promising target for the development of new drugs since: (A) no homologs of the permease have been found in mammals; (B) is responsible for the intracellular availability of essential metabolites, (C) regulates many metabolic pathways and parasite stress responses, (D) mediates the uptake of trypanocidal drugs, (E) is essential to sustain the parasite infection (11), and (F) inhibition of polyamine transport by drugs has a strong trypanocidal effect (12–16).

In cancer cells, high polyamine concentrations are required by every cell stage. Besides polyamine biosynthesis, cancer cells also utilize polyamine importers to augment their intracellular polyamine pools. These transporters can be targeted via the delivery of cytotoxic polyamine conjugates or via drugs which inhibit polyamine uptake (17). The co-administration of polyamine transport and biosynthesis inhibitors was successfully tested in cancer models. For example, the use of benzene derivatives of polyamines in combination with the ornithine decarboxylase inhibitor, difluoromethylornithine (DFMO), produced a cytotoxic effect in Chinese hamster ovary cells (CHO) and L3.6pl human pancreatic cancer cells (18). In addition, similar effects were observed in CHO cells using polyamines conjugated with the cytotoxic anthracene (19).

Prior work with a 9-anthracenylmethyl-putrescine conjugate (Ant4) explored its ability to inhibit polyamine transport and affect cell viability. Ant4 induced cytotoxicity in the HL-60 cell line after only 24 h exposure with an IC_50_ of 20 μM, and apoptosis was the main mechanism of cell death. Ant4 was shown to inhibit putrescine transport and decreased its intracellular concentration (17). This conjugate was tested not only in mammalian cells, but also in unicellular parasites. For example, in the human malaria parasite *Plasmodium falciparum*, Ant4 inhibited the proliferation of the intraerythrocytic stage with an IC_50_ of 0.64 μM (20).

In our previous studies with *T. cruzi*, Ant4 decreased the putrescine and spermidine transport in epimastigotes, the insect stage of the parasite, with IC_50_ values of 5.2 and 8.8 μM, respectively. Ant4 also showed a strong trypanocidal effect on trypomastigotes, the bloodstream stage of *T. cruzi*, with an IC_50_ of 460 nM, and a selectivity index of about 13. This trypanocidal effect of Ant4 is very promising since it is significantly higher than the observed for benznidazole, the drug currently used to treat Chagas disease. In addition, the combination of both drugs produced a significant increase on the trypanocidal effect compared with individual treatments (14).

One of the strategies applied to identify drugs for neglected diseases is the search for approved compounds used in treating other pathologies. This strategy is known as drug repurposing or drug repositioning. One of the main advantages of this experimental approach is that reduces the time and the economic cost necessary to apply approved drugs in the treatment of other diseases. This is especially relevant in orphan diseases, like Chagas disease, which have only few drug alternatives available. Taking into account the high trypanocidal activity of Ant4, and that this molecule is not approved for use in humans, in this work we identified, using *in silico* and *in vitro* strategies, three antipsychotic tricyclic drugs which have similar structure and activity to Ant4.

## Materials and Methods

### Parasites and Cells

*Trypanosoma cruzi* epimastigotes of the Y strain (5 × 10^6^ cells/mL) were cultured at 28°C in plastic flasks (25 cm^2^), containing BHT (brain-heart infusion-tryptose, 5 mL) medium supplemented with 10% fetal calf serum (FCS), 100 U/mL penicillin, 100 μg/mL streptomycin and 20 μg/mL hemin. Vero cells (African green monkey kidney) were cultured in MEM medium supplemented with 10% heat inactivated FCS, 0.15% (w/v) NaHCO_3_, 100 U/mL penicillin and 100 U/mL streptomycin at 37°C in 5% CO_2_ atmosphere. Trypomastigotes and amastigotes of the Y strain were obtained from Vero infected cells as previously described (21).

### Transport Assays

Aliquots of epimastigote or trypomastigote cells were centrifuged at 8,000 g for 30 s, and washed once with phosphate-buffered saline (PBS). Parasites were resuspended in PBS (0.1 mL) and the assay started by the addition of 0.1 mL of the transport mixture containing [^3^H]-putrescine or [^3^H]-spermidine (PerkinElmer's NEN^®^ Radiochemicals; 0.4 μCi) in the presence of different concentrations of the indicated drug. All the compounds to be tested were dissolved in water. Following incubation during 10 min at 28°C (epimastigotes) or 37°C (trypomastigotes), transport was stopped by adding 1 mL of ice-cold PBS. Cells were centrifuged as indicated above, and washed twice with ice-cold PBS. Cell pellets were resuspended in 0.2 mL of water and counted for radioactivity in UltimaGold XR liquid scintillation cocktail (Packard Instrument Co., Meridien CT, USA). Cell viability was assessed by direct microscopic examination. Non-specific uptake and carry over were assayed without incubation or incubated at 4°C (22).

### Trypanocidal Activity Assays

*T. cruzi* epimastigotes were cultured as described above, in 24-wells plate at a start density of 10^6^ cells/mL in BHT medium. Parasites were treated with different concentrations of each drug, and epimastigote proliferation was determined after 24 h. Trypanocidal activity in cell-derived trypomastigotes and amastigotes was performed using 10^6^ cells/mL in 96-well plates which were incubated at 37°C for 24 h in the presence of the corresponding drug. Growth was determined by counting in a Neubauer chamber or via viability assays using “Cell Titer 96^®^ Aqueous One Solution Cell Proliferation Assay (MTS)” (Promega, Madison, WI, USA) according to the manufacturer's instructions.

### Cell Viability Assay

Cytotoxicity against VERO cells was determined by the crystal violet staining assay. The cells (10^4^ cells/well) were incubated in 96-well plates with the indicated compound (or diluent only as a negative control) and maintained at 37°C for 24 h. At the end of treatment, cells were fixed for 15 min, and stained with 0.5% crystal violet. After washing with water and drying, the absorbance of stained cells was measured at 570 nm.

### Intracellular Localization of Ant4 and Chlorpromazine by Fluorescence Microscopy

Epimastigotes (2 × 10^6^ cells/ml) were incubated with the corresponding concentrations of each compound for 30 min at 28°C. Afterwards, the samples were settled for 20 min at room temperature onto poly-L-lysine coated coverslips and then fixed for 20 min with 2% paraformaldehyde in PBS. Slides were mounted using 80% glycerol in PBS and cells were observed under an Olympus BX60 fluorescence microscope. Images were recorded with an Olympus XM10 camera.

### Virtual Screening

Similarity screening searches were performed using the 9-anthracenylmethyl-putrescine conjugate, Ant4, as the reference (query) compound. Structures (≈10,000) were obtained from the highly curated “Sweetlead” database of the world's approved medicines, illegal drugs, and isolates from traditional medicinal herbs (23). The screening was performed using the software LiSiCA v1.0 (Ligand Similarity using Clique Algorithm) (24). Different control structures were used in screening searches, in order to check if they were identified with higher scores in each resulting list, thus validating the correct operation of the program. Shared chemical features between structures were identified by feature-based structure alignments using the LigandScout algorithm (25).

### Docking Simulations

The tested molecules as well as the natural ligands of TcPAT12, putrescine, and spermidine, were used to model the mode of interaction with an homology model of the transporter using the molecular docking algorithm in AutoDock 4.5 (26) as described previously (16). For docking simulations, a grid that covers the whole transporter molecule was applied and the program was run using a Lamarckian Genetic Algorithm 100 times, with a population size of 300, and 2.7 × 10^4^ as maximum number of generations. The figures of the docking poses where produced using Pymol (The PyMOL Molecular Graphics System, Version 2.0 Schrödinger, LLC.) and the protein residues important for the interaction with the ligands were colored using Pymol according to the Eisenberg hydrophobicity scale (27).

### Statistics and Data Analysis

IC_50_ and TIC_50_ values were obtained by non-linear regression of dose-response logistic functions, using GraphPad Prism 6.01 for Windows, and the corresponding R-square was indicated for each curve. All experiments were performed in triplicate and the data are presented as mean ± standard deviation (SD). *P*-values of the comparisons were calculated using the extra sum of squares *F*-test.

## Results

### Similarity Virtual Screening

**Figure 1:**
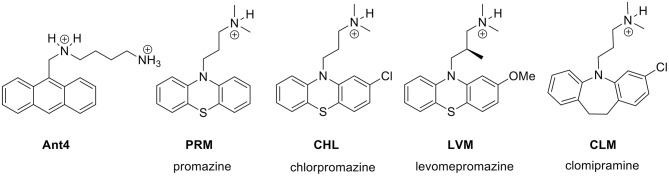
Structures of Ant4 chemical analogs obtained by similarity-based virtual screening. The antipsychotic tricyclic drugs, promazine (PRM), chlorpromazine (CHL), levomepromazine (LVM) and clomipramine (CLM) were selected for *in vitro* assays in *T. cruzi* parasites.

Ant4 is an experimental drug for cancer treatment not yet approved for use in humans, which also has a strong trypanocidal action (14). Therefore, the first step to identify an approved drug with similar activity to Ant4 was to perform a similarity-based virtual screening with the final objective of repurposing the resulting drugs as trypanocidal agents. Ant4 was defined as the query molecule to compare with 10,000 chemical structures of the Sweetlead database using the LiSICA software. Applying this strategy, four drugs were chosen for further analysis between the top 10 scored compounds with a Tanimoto coefficient (TC, a structural similarity index) > 0.5, and also considering their availability, price and toxicity. These drugs were promazine (PRM; ZINC ID: 10402; TC = 0.64), chlorpromazine (CHL; ZINC ID: 44027; TC = 0.58), levomepromazine (LVM; ZINC ID: 20246; TC = 0.58), and clomipramine (CLM; ZINC ID: 20248; TC = 0.56). Interestingly, all of them are antipsychotic tricyclic drugs (Figure 1). The LigandScout algorithm was used to identify common chemical features (groups that can participate in chemical interactions with a macromolecule) between Ant4 and the obtained drugs. Feature-based structure alignments were performed and the similarities were calculated as the number of matched feature pairs (MFP; i.e., aromatic ring, hydrophobic area, hydrogen bond donor or acceptor, negative or positive ionizable atom, and metal binding location). For these comparisons, the ten features present in Ant4 were set as references. CHL was the structure that shares more chemical features with Ant4 (5 out of 10); including three hydrophobic interactions, one hydrogen bond donor and one positive ionizable interaction. Alignment results are shown in Supplementary Figures S1, S2.

### Inhibition of Polyamine Transport by Ant4 Chemical Analogs

**Figure 2:**
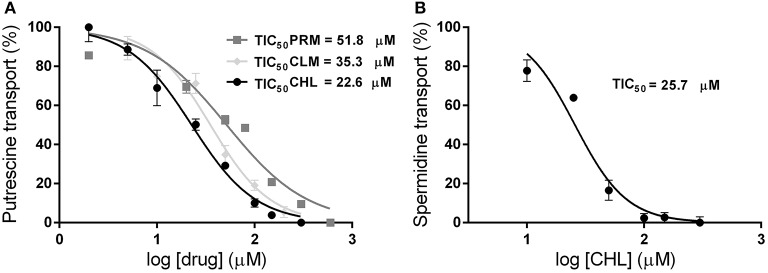
Effect of promazine, chlorpromazine and clomipramine on polyamine transport in *T. cruzi* epimastigotes. Transport assays were performed incubating 10^7^ epimastigotes with 5 μM [^3^H]-putrescine **(A)** or 15 μM [^3^H]-spermidine **(B)** in the presence of different concentrations of drugs (PRM, dark gray squares; CLM, light gray diamonds; CHL, black circles) between 0 and 600 μM. The concentrations that inhibit the polyamine transport in a 50% (TIC_50_) were calculated by non-linear regression using the GraphPad Prism software ($R_{\text{PRM}}^{2}$ = 0.945; $R_{\text{CLM}}^{2}$ = 0.962; $R_{\text{CHL}}^{2}$ = 0.981 for putrescine, $R_{\text{CHL}}^{2}$ = 0.951 for spermidine).

To test the similarity screening predictions, the ability of PRM, CHL, LVM, and CLM to inhibit the polyamine uptake was analyzed. Transport assays were performed in epimastigote cells in the presence of putrescine or spermidine (5 and 15 μM, respectively) and each putative inhibitor in a concentration range of 0–600 μM. Concentrations that inhibit the 50% of the transport activity (TIC50) were calculated. PRM, CHL, and CLM produced an inhibition of putrescine transport with calculated TIC50 values of 51.8 μM (±4.7), 22.6 μM (±1.3), and 35.3 μM (±2.6), respectively (Figure 2A). However, the spermidine transport was inhibited only by CHL with a calculated TIC50 of 25.7 μM (±1.9) (Figure 2B). LVM did not produce an inhibition of polyamine transport in this concentration range. No alterations in the parasites viability were observed after the transport assays indicating that the inhibitory effect was not due to compounds toxicity.

### Trypanocidal Effect of Ant4 Analogs

**Figure 3:**
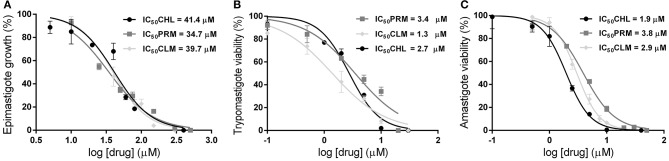
Trypanocidal effect of Ant4 chemical analogs on different stages of the *T. cruzi* life cycle. 10^6^ exponential phase epimastigotes were treated with different concentrations of drugs (PRM, dark gray squares; CLM, light gray diamonds; CHL, black circles) between 0 and 500 μM and incubated for 24 h **(A)**. Culture-derived trypomastigotes **(B)** or amastigotes **(C)** (10^6^ cells/mL) were exposed to compounds for 24 h in the concentration range of 0–50 μM **(B)**. Inhibitory concentrations for 50% parasites after 24 h (IC_50_) were carried as described in the Materials and Methods section ($R_{\text{PRM}}^{2}$ = 0.973; $R_{\text{CLM}}^{2}$ = 0.977; $R_{\text{CHL}}^{2}$ = 0.923 for epimastigotes; $R_{\text{PRM}}^{2}$ = 0.934; $R_{\text{CLM}}^{2}$ = 0.966; $R_{\text{CHL}}^{2}$ = 0.973 for trypomastigotes; $R_{\text{PRM}}^{2}$ = 0.992; $R_{\text{CLM}}^{2}$ = 0.995; $R_{\text{CHL}}^{2}$ = 0.988 for amastigotes).

The trypanocidal activity was evaluated only for those drugs that inhibited polyamine transport activity, namely PRM, CHL, and CLM. Interestingly, all these compounds presented trypanocidal effect against the three stages of *T. cruzi*. Epimastigotes were treated with each drug in concentrations between 0 and 500 μM and the trypanocidal effect was determined 24 h post-treatment. The calculated concentrations that inhibit the 50% of the parasite proliferation (IC_50_) were 34.7 μM (±2.3), 41.4 μM (±4.0), and 39.7 μM (±2.8) for PRM, CHL, and CLM, respectively (Figure 3A). Next, the trypanocidal activity of these drugs was also assessed in cell-derived trypomastigotes and amastigotes in concentrations between 0 and 50 μM and IC_50_ values were calculated 24 h post-treatment. PRM, CHL and CLM showed similar trypanocidal effects on both parasite forms and presented significantly lower IC_50_ values than those obtained for the epimastigote stage (*p* < 0.0001 for all drugs); corresponding to 3.4 μM (±0.5), 2.7 μM (±0.2), and 1.3 μM (±0.2) for trypomastigotes cells and 3.8 μM (±0.2); 1.9 μM (±0.1), and 2.9 μM (±0.1) for amastigote parasites (Figures 3B,C). This means that *T. cruzi* trypomastigotes and intracellular amastigotes, the therapeutically relevant stages present in the mammalian host, are about 10 to 20-fold (depending on the drug) more susceptible than epimastigotes, the insect stage of the parasite. Additionally, the cytotoxicity of drugs was tested using mammalian Vero cells to determine the selectivity index (SI = IC_50_ on Vero cells/IC_50_ on trypomastigotes). Cells exposed to PRM, CHL, and CLM for 24 h in a concentration range from 0 to 300 μM showed SI values of 17.9 (IC_50_ 62.8 μM ± 3.6), 9.4 (IC_50_ 26.4 μM ± 0.3), and 38.2 (IC_50_ 53.5 μM ± 2.1), respectively. These results indicate that the drugs are more selective against *T. cruzi* trypomastigotes than host cells.

### Effect of Ant4 Analogs on Polyamine Transport in *T. cruzi* Trypomastigotes

In order to further test the correlation between the trypanocidal activity of PRM, CHL, and CLM and the polyamine transport, the inhibitory effect of the drugs on putrescine and spermidine uptake in the trypomastigote stage of *T. cruzi* was also evaluated. PRM, CHL, and CLM were tested at concentrations similar to the TIC50 values calculated on epimastigotes. As occurred with epimastigotes, all the compounds inhibited the putrescine transport in trypomastigotes. The results showed that 50 μM PRM, 25 μM CHL, and 35μM CLM produced inhibition of 83% (±2.2), 84.3% (±5.9), and 79.2% (±11.4) respectively. In addition, in this stage of the parasite all drugs also inhibited the spermidine transport. At 25 μM the inhibitions observed were 64.6% (±2.7), 55.7% (±0.7), and 60.2% (±9.5) for PRM, CHL, and CLM, respectively. These results suggest that the compounds evaluated act through a polyamine transport inhibition mechanism in *T. cruzi* epimastigotes and trypomastigotes.

### Binding Properties of Ant4 and Its Chemical Analogs

**Figure 4:**
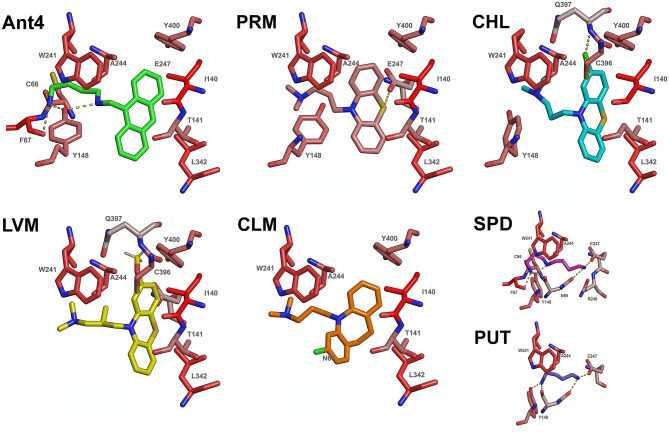
Docking simulations. The docking poses and the residues important for each interaction are shown, where the dashed yellow lines represent the polar contacts and possible hydrogen bonds between the transporter and the ligands. Note: the residues are colored according to the Eisenberg hydrophobicity scale (27) where increased red color denotes higher hydrophobic character.

Using a previously reported homology-modeled structure of the *T. cruzi* polyamine permease TcPAT12 (16), the ability of Ant4, PRM, CHL, LVM, and CLM to bind the transporter was tested by a computer-assisted simulation method. AutoDock software was used to calculate possible conformations of the ligands which bind TcPAT12. Interestingly, the ranking of the docking scores correlates with the ranking of transport inhibition as presented in Table 1. The docking poses with the residues involved in the interaction of each compound with TcPAT12 are schematized in Figure 4. In all the ligands, the aliphatic carbon chain occupies a hydrophobic site formed by residues W241 and A244. In the docking simulations, the putrescine motif in putrescine, spermidine, and Ant4 fell in the same position of the pocket. The polycycle of Ant4 occupied a hydrophobic pocket formed by Y400, I140, T141, Y148, and A244. The ring structures in PRM and its analogs occupy the same hydrophobic pocket as Ant4. Between the promazine derivatives, CHL differs from PRM just in its chlorinated polycycle, and this accounts for the stronger inhibitory effect of CHL. LVM docking pose is very similar to that of CHL, but the methoxy group (that substitutes the Cl) clashes with the protein in a very hydrophilic pocket, which probably explains why LVM was not able to inhibit the transporter experimentally. CLM had a docking pose very similar to all the other compounds, but with the chloride in the opposite side of the pocket. We hypothesize that the additional carbon atoms in the central ring orients the chloride at an angle that makes it unable to occupy the same position as in CHL.

**Table 1:** Predicted binding energies by molecular docking of the tricyclic drugs.

**Compound**	**ZINC ID**	**dG (mean)**	**dG (lower)**
Ant4	N/A	−8.38	−9.90
Clomipramine	20248	−7.35	−8.29
Chlorpromazine	44027	−7.29	−8.38
Promazine	10402	−6.64	−7.64
Levomepromazine	20246	−6.27	−7.64
Putrescine^*^	5828633	−2.42	−3.31
Spermidine^*^	1532612	−2.17	−3.33

All tricyclic drugs obtained by similarity searching, in addition to the reference compound Ant4 and the substrates of TcPAT12

(*), *were listed in the table. Columns indicate the compound, the ID according to the ZINC database (http://zinc.docking.org/), the mean (dG mean), and the lower (dG lower) ligand efficiencies of the most populated cluster. Those compounds that produced a significant inhibition of polyamine transport and trypanocidal activity were highlighted (light gray). Ligand efficiency values were calculated using the AutoDock program*.

### Incorporation of Ant4 and Chlorpromazine

**Figure 5:**
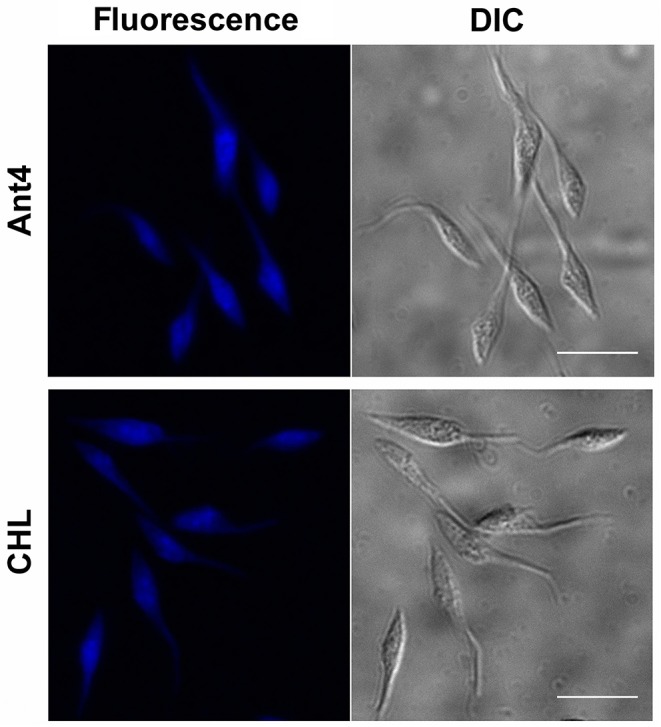
Intracellular localization of Ant4 and chlorpromazine in *T. cruzi*. Fluorescence microscopy images and corresponding differential interference contrast images (DIC) of epimastigotes treated with 50 μM Ant4 or 25 μM CHL for 30 min. Scale bar = 10 μm.

To test whether the tricyclic drugs studied not only block the transport of polyamines but also are incorporated into the cell, the intrinsic fluorescence signal of Ant4 and CHL was used as an analysis tool. When the accumulation of these compounds into cells was visualized by fluorescence microscopy, epimastigotes treated with 50 μM Ant4 or 25 μM CHL for 30 min showed that these drugs present very similar pattern of intracellular localization, dispersed through the cytoplasm of the parasites (Figure 5). These results, together with those obtained in transport assays, demonstrate that both Ant4 and CHL are actively transported into the *T. cruzi* cell.

## Discussion

Tricyclic neuroleptic drugs, including clomipramine and chlorpromazine, were previously described as therapeutic agents for trypanosomiasis. The most studied mechanism of action was the inhibition of the trypanothione reductase; the enzyme responsible for the synthesis of the antioxidant compound trypanothione, the parasite analog of glutathione (28, 29). However, other properties and multi-target effects were reported, among them are: (a) the inhibition of the enzyme dihydrolipoamide dehydrogenase (30), (b) the interaction with nucleic acids, and (c) the generation of phenothiazines cation radicals. In this work we describe a novel mechanism of action of these drugs in *Trypanosoma cruzi*; the inhibition of the polyamine transporter TcPAT12 in addition to the possible intracellular toxic effects. Using the experimental oncologic drug Ant4 (not approved for use in humans) which has a strong activity on *T. cruzi* we performed a similarity virtual screening and identified three antipsychotic drugs; promazine, chlorpromazine, and clomipramine, which were repositioned as trypanocidal agents. All parasite stages tested for trypanocidal activity were affected by Ant4 and the tricyclic drugs herein reported and an increased sensitivity of the trypomastigote and amastigote stages was observed. It should be noted that psychiatric patients, who receive oral treatments with chlorpromazine or clomipramine within the reference doses, reach plasma concentrations in the range of the calculated IC_50_ for both drugs in the stages present in the mammalian host. In consequence these medicines are promising candidates to perform pre-clinical evaluations for the development of new therapeutic alternatives to treat Chagas disease.

The differential trypanocidal effect of polyamine transport inhibitors on *T. cruzi* epimastigote and trypomastigote stages was previously observed by our group (14, 16) and could be explained due to the variability of polyamine concentrations in the niche of each stage. For example, the epimastigote stage supports a highly variable extracellular media within the intestine of the vector and depends on the feeding status of the insect. In contrast, the trypomastigote stage lives in a medium with scarce fluctuations within the mammalian host. Therefore, it is likely that the trypomastigotes have less efficient compensatory mechanisms to support a decrease in the concentration of polyamines caused by the TcPAT12 inhibitors. The fact that the effect of Ant4 and its analogs is higher in the non-replicative trypomastigote stage also suggests that the main mechanism of toxicity is on the transport of polyamines and not on cell division, where anthracene would also act as a DNA intercalating agent. This sensitivity difference between the stages of the *T. cruzi* life cycle is important since the trypomastigote is the most sensitive stage and the most relevant from a therapeutic point of view.

Other trypanocidal drugs that inhibit the polyamine transport mediated by TcPAT12 were identified by virtual screening techniques. Isotretinoin, a popular drug used for acne treatment, showed the most stable predicted interaction with TcPAT12 and inhibited the polyamine transport *in vitro*. Moreover, isotretinoin showed a strong inhibition of trypomastigote burst from infected cells with a calculated IC_50_ of 130 nM and a selectivity index of 920 and is a promising drug for pre-clinical trials (16).

Fluorescence microscopy studies also revealed the uptake and intracellular accumulation of Ant4 and chlorpromazine. Both compounds were internalized by *T. cruzi* epimastigotes suggesting distinct cellular targets besides polyamine transport. In human cancer cells it has been described that Ant4 anthracene moiety interact and induces DNA damage (17), however, as previously mentioned, no correlation between replication and toxicity was observed. In this sense, further studies are required to identify these targets in the parasite.

Our data suggests that both the putrescine moiety and the large hydrophobic ring of Ant4 contribute to its polyamine transport inhibition activity. From the perspective of designing new TcPAT12 inhibitors, the docking models suggest that these compounds should keep the putrescine motif, while retaining all protonable nitrogen as in the case of Ant4. Moreover, large hydrophobic rings should be present to anchor the drug into the hydrophobic pocket previously described. This work also suggests that halogen substitution on the hydrophobic moiety could provide higher binding affinity for the transporter.

All these results highlight the importance of the polyamine transporter TcPAT12 as a therapeutic target for Chagas disease and drug repositioning as an adequate strategy for drug discovery to treat neglected diseases.

## Data Availability Statement

All datasets generated for this study are included in the article/Supplementary Material.

## Author Contributions

CR and CP conceived and designed the experiments. CR, MS, EV-V, and MM performed the experiments. CR, OP, MS, EV-V, MM, and CP analyzed and interpreted the data. CP and OP contributed reagents, materials, analysis tools, or data. CR, CP, and OP wrote the paper. CR, OP, MS, EV-V, MM, and CP revised the manuscript.

### Conflict of Interest

The authors declare that the research was conducted in the absence of any commercial or financial relationships that could be construed as a potential conflict of interest.
