# Supplementary material for: Identification of Trypanosoma cruzi Polyamine Transport Inhibitors by Computational Drug Repurposing
Source: Front Med (Lausanne). 2019 Nov 8;6:256. doi: 10.3389/fmed.2019.00256 (PMC6857147; doi:10.3389/fmed.2019.00256)
Supplement: Supplementary file 1 [file Data_Sheet_1.PDF]

Supplementary Figure S1.

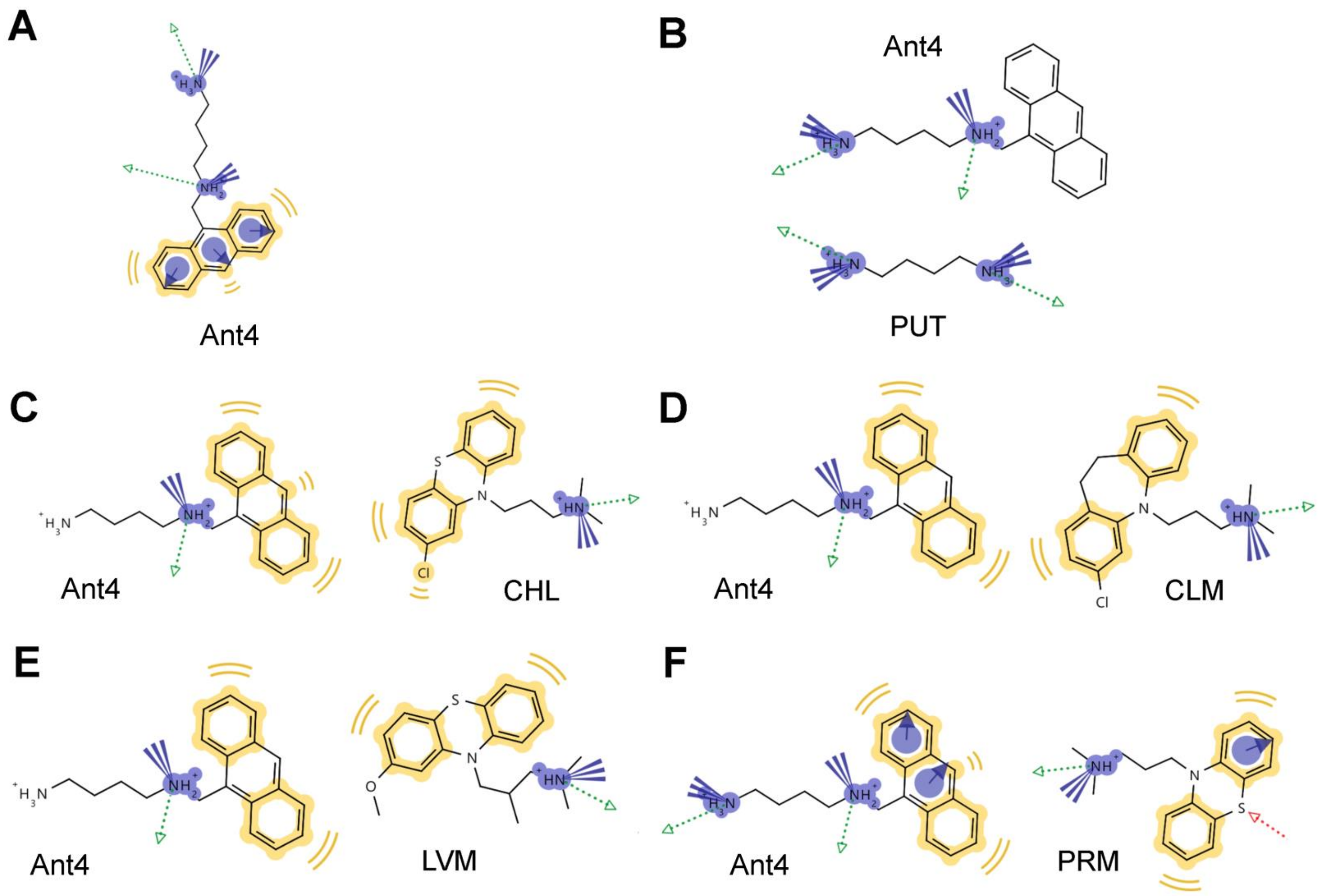

Chemical features shared between Ant4 and its chemical analogues. In order to perform the structure comparisons using the LigandScout software, the 10 Ant4 features (A) were set as references and the features shared with putrescine (B), chlorpromazine (C), clomipramine (D), levomepromazine (E), and promazine (F) were schematized. Chemical features: aromatic ring (purple circles with arrow); hydrophobic area (orange waves with ring remarks); positive ionizable atom (purple lines triplets); H-bond donor (dotted line green arrow); and H-bond acceptor (dotted line red arrow).
