# Supplementary material for: Identification of Trypanosoma cruzi Polyamine Transport Inhibitors by Computational Drug Repurposing
Source: Front Med (Lausanne). 2019 Nov 8;6:256. doi: 10.3389/fmed.2019.00256 (PMC6857147; doi:10.3389/fmed.2019.00256)
Supplement: Supplementary file 2 [file Data_Sheet_2.PDF]

Supplementary Figure S2.

Compound	Score	MFP	RMS
Ant4	109	10	0
chlorpromazine	55.4	5	1.2
clomipramine	45.4	4	1.2
levomepromazine	45.4	4	1.2
promazine	45.3	4	1.2

The LigandScout software was used to identify the common chemical features between Ant4 and its chemical analogues. This algorithm performs feature-based structure alignments where the similarities are calculated as the number of matched feature pairs (MFP). Columns showed the LigandScout similarity score (Score), the number of MFP obtained for each compound (MFP) and the root mean square (RMS) of their positions.
